# Posterior cortical atrophy as a primary clinical phenotype of corticobasal syndrome with a progranulin gene rs5848 TT genotype

**DOI:** 10.1186/s13023-016-0396-0

**Published:** 2016-02-06

**Authors:** Guoping Peng, Ping Liu, Fangping He, Benyan Luo

**Affiliations:** Department of Neurology, First Affiliated Hospital, College of Medicine, Zhejiang University, 79 Qingchun Road, Hangzhou, 310003 China

**Keywords:** Progranulin, rs5848, Posterior cortical atrophy, Corticobasal syndrome

## Abstract

Posterior cortical atrophy (PCA) represents a special clinicoradiologic syndrome characterized by progressive visuospatial and visuoperceptual deficits. PCA and corticobasal syndrome (CBS) may share similar pathogenetic mechanisms. We report the clinical, neuropsychological, imaging, and genetic features of a patient with initial visual problems, who further developed other cognitive impairments and asymmetric extrapyramidal signs fitting into the diagnosis of CBS. Genetic testing revealed homozygous for the T allele of the rs5848 GRN variant. This study provided an evidence for CBS belonging to the clinical spectrum of GRN genetic variant and demonstrated CBS may initially present with symptoms of PCA in rare cases.

## Correspondence

Letters to the Editor:

Posterior cortical atrophy (PCA) is a neurodegenerative disorder characterized by progressive visuospatial and visuoperceptual deficits [1]. The dynamics of its progression and clinical outcome are not well known. Pathological studies have shown that Alzheimer’s disease (AD) is the most common underlying etiology of PCA. However, a small number of cases are also reported with other degeneration diseases [2]. The exact genetic basis of PCA remains unclear, however, mutations have been reported in the prion protein gene (*PRNP*) [3], presenilin 1 and 2 genes (*PSEN1* and *PSEN2*) [4], microtubule-associated protein tau gene (*MAPT*) [5], and progranulin gene (*GRN*) [6], suggesting heterogeneity of its genetic mechanism. Here, we describe a case of a PCA patient with initial visual problems, who developed further cognitive impairments and asymmetric extrapyramidal signs that fitted with a diagnosis of corticobasal syndrome (CBS).

**Fig. 1:**
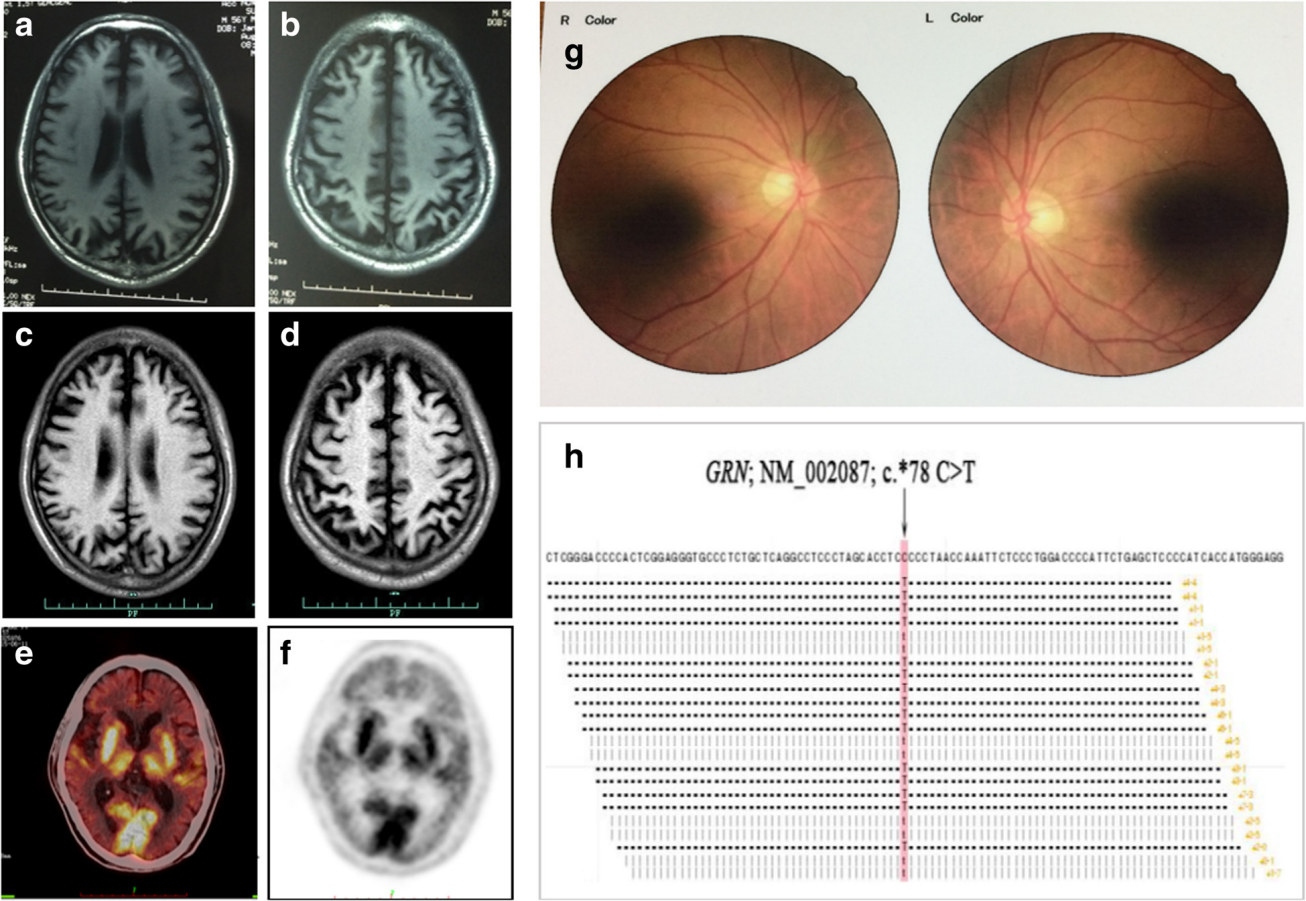
The clinical materials of the case. **a**-**b** Axial sections, T1-weighted sequences of MRI 4 years after disease onset (at 56 years of age) revealed cerebral atrophy, particularly in the posterior cortex. **c**-**d** One and a half years later, the secondary MRI revealed much more extensive atrophy within the frontal and occipital-parietal lobes, and bilateral partial parietal lobe. **e**-**f** 18F-FDG-PET showed diffuse bilateral hypometabolism in the frontal, parietal, temporal, and occipital lobes, particularly in the parieto-occipital regions. **g** Bilateral fundus examinations were normal. **h** Genetic testing showed a single nucleotide polymorphism rs5848 (c.*78C > T), located in the 3′-UTR of *GRN*

The patient’s initial symptoms were discovered in early 2009 at the age of 52, as it was noted that he took longer time to return home alone than before. He also began to complain of blurred vision, but it did not affect his daily life. Two years later, he began to suffer from progressive spatial disorientation. He was unable to fetch objects with either arm by visual guidance. His memory also began to decline and sometimes he spoke not fluently. In 2012, he exhibited problems in writing simple words, calculating and discerning fingers. He walked more slowly and sometimes exhibited tremor and myoclonus of his upper limbs, especially his right side. In May 2013, he complained of deterioration and difficulty in tracking specific objects in his visual field. He also had difficulty in riding, dressing himself, and distinguishing coats from pants. He urinated anywhere as he could not find the bathroom, and he even occasionally defecated on himself. Structural brain magnetic resonance imaging (MRI) in a local hospital revealed cerebral atrophy, particularly in the posterior cortex (Fig. 1a, b). He was diagnosed with AD. After 6 months, he stopped donepezil treatment since his symptoms got more severe.

Positive neurological system signs included reduced facial expression, increased muscle tone and bradykinesia especially on the right side and a little resting tremor on his right side. His performance in neuropsychological evaluations (Table 1) suggested that he had problems in visual spatial, memory, language, and motor functions. He had no significant past medical history and no family history of dementia.

**Table 1:** Neuropsychological profile of the patient

Tests	Patient’s score/Maximal score
Mini-mental State Examination (MMSE) scores	6/30
Time and location (orientation)	1/10
Immediate recall	1/3
Repetition	1/1
Naming	2/2
Executive function	1/3
Montreal Cognitive assessment Scale (MoCA) Scores	Unfinished (noncooperation)
Boston naming test (30 items)	6/30
Clock drawing test	0/4
Forward digit-span task	4
Backward digit-span task	2
Plane copy	0
Three dimensional copy	0
Rey complex figure test	0
Trail making test A	Unfinished
Trail making test B	Unfinished
Hamilton Anxiety Scale (HAMA)-17 items	25/68
Hamilton Depression Scale (HAMD)-17 items	24/68
Activities of Daily living (ADL) scale-14 items	48/64
Hachinski Ischemic Scale (HIS)	3/18

Secondary MRI revealed marked cortical atrophy within the frontal and occipito-parietal lobes, and partial atrophy in the bilateral parietal lobes (Fig. 1c, d). 18F-fluorodeoxyglucose positron emission tomography (18F-FDG-PET) showed diffuse bilateral hypometabolism in the frontal, parietal, temporal, and occipital lobes, particularly in the occipito-parietal regions (Fig. 1e, f). Ophthalmology examination found that his fundus (Fig. 1g) and intraocular pressure were normal. His visual evoked potential was nearly normal, while his visual field and visual acuity examinations were not completed because of noncooperation. A laboratory blood screening test for reversible dementia was unremarkable. Lumber puncture showed normal cerebrospinal fluid (CSF) pressure, glucose, chlorine, cell number, and protein levels. Normal total-tau, phosphorylated-tau, and amyloid-β (Aβ) 1–42 levels were also reported in CSF. No pathogenic mutations in early-onset AD-associated genes (including *APP*, *PS1*, *PS2*, *BACE1*, and *PRNP*) were found. The patient’s *APOE* genotype was E3/E3. However, testing for frontotemporal lobar degeneration (FTLD) -associated gene (*GRN*, *CHMP2B*, *FUS*, *MAPT*, *PSEN1*, *TARDBP* and *VCP*) mutations revealed a T allele of rs5848, locating in the 3′-UTR of *GRN* (Fig. 1h).

## Discussion

Posterior cortical atrophy is a rare clinicoradiologic neurodegenerative syndrome, and two sets of clinical diagnostic criteria for PCA have been proposed [7, 8]. The core features of disease onset, progress forms, and main clinical manifestations are consistent, but do not illustrate its underlying pathological, genetic, or biomarker standards.

In the initial phase of our case, the symptoms were visual problems, specifically, of disorientation and blurred vision. Over time he manifested additional symptoms including optic ataxia, oculomotor apraxia, right-left disorientation, acalculia, dressing apraxia, and agraphia, fitting into the dorsal subtype of PCA. His first cerebral MRI revealed posterior cortical atrophy, also supporting his initial clinical diagnosis. As the disease progressed, the patient developed further asymmetric extrapyramidal signs and progressive global cognitive impairment, but without vertical ophthalmoplegia or apparent hallucinations. The second MRI revealed much more extensive cerebral atrophy and FDG-PET confirmed hypometabolism of those cortical regions. Given the history, clinical findings, and supporting examinations, a diagnosis of CBS was then made [9].

Besides corticobasal degeneration, CBS can also be caused by such as progressive supranuclear palsy, AD, Pick’s disease, FTLD with TDP-43 inclusions, mutations in *GRN* or *MAPT*, DLB, and CJD [10]. Considering current clinical criteria and various pathological etiologies for PCA and CBS, there is considerable overlap, meaning that some patients may fulfill criteria for both syndromes, or develop from one syndrome to the other [11].

Special biomarkers such as the CSF tau:Aβ ratio may predict pathology in CBS patients [12]. In our case, CSF analysis revealed normal tau and Aβ1-42 levels, which suggests low probability of AD pathology. In addition, genetic tests identified no reported pathogenic genetic mutations for early-onset AD, and an *APOE* E3/E3 genotype. FTLD-associated gene testing revealed a genetic variant, rs5848 (c.*78C > T) in the 3′-UTR of the *GRN* gene, which has been reported as a risk factor for TDP-43-positive frontotemporal dementia and other neurodegenerative diseases such as AD. The *GRN* rs5848 TT genotype is reported to improve microRNA binding efficiency to the 3′-UTR of *GRN*, leading to enhanced suppression of GRN translation and reduced GRN expression [13]. Tartaglia et al. [14] reported a typical patient with sporadic CBS, who was homozygous for this *GRN* variant (rs5848), and without mutations in *MAPT* or *GRN* genes. Recently, Caroppo et al., [6] reported a visual/ventral variant in a PCA patient carrying a heterozygous *GRN* mutation. Whether this PCA patient would develop other symptoms that meet the diagnosis of CBS, like our case, needs longitudinal follow-up and final autopsy evidence.

This study provides evidence for CBS belonging to the *GRN* genetic variant (rs5848) clinical spectrum, and demonstrates that in rare cases, CBS may initially present with symptoms of PCA. Longitudinal follow-up is required to ascertain the most likely etiology and determine the clinical-genetic-pathological mechanism of the T allele of the rs5848 polymorphism in CBS diagnosis.

## Abbreviations

PCA: Posterior cortical atrophy
AD: Alzheimer’s disease
DLB: Dementia with Lewy bodies
PRNP: Prion protein gene
MAPT: Microtubule-associated protein tau gene
GRN: Progranulin gene
CBS: Corticobasal syndrome
MRI: Magnetic resonance imaging
18F-FDG-PET: 18F-fluorodeoxyglucose positron emission tomography
CSF: Cerebrospinal fluid
FTLD: Frontotemporal lobar degeneration

## References

[1] Tsai PH, Teng E, Liu C, Mendez MF (2011). Posterior cortical atrophy: evidence for discrete syndromes of early-onset Alzheimer’s disease. Am J Alzheimers Dis Other Demen.

[2] Crutch SJ, Lehmann M, Schott JM, Rabinovici GD, Rossor MN, Fox NC (2012). Posterior cortical atrophy. Lancet Neurol.

[3] Depaz R, Haik S, Peoc’H K, Seilhean D, Grabli D, Vicart S (2012). Long-standing prion dementia manifesting as posterior cortical atrophy. Alzheimer Dis Assoc Disord.

[4] Sitek EJ, Narozanska E, Peplonska B, Filipek S, Barczak A, Styczynska M (2013). A patient with posterior cortical atrophy possesses a novel mutation in the presenilin 1 gene. PLoS One.

[5] Rossi G, Bastone A, Piccoli E, Morbin M, Mazzoleni G, Fugnanesi V (2014). Different mutations at V363 MAPT codon are associated with atypical clinical phenotypes and show unusual structural and functional features. Neurobiol Aging.

[6] Caroppo P, Belin C, Grabli D, Maillet D, De Septenville A, Migliaccio R (2015). Posterior cortical atrophy as an extreme phenotype of GRN mutations. JAMA Neurol.

[7] Mendez MF, Ghajarania M, Perryman KM (2002). Posterior cortical atrophy: clinical characteristics and differences compared to Alzheimer’s disease. Dement Geriatr Cogn Disord.

[8] Tang-Wai DF, Graff-Radford NR, Boeve BF, Dickson DW, Parisi JE, Crook R (2004). Clinical, genetic, and neuropathologic characteristics of posterior cortical atrophy. Neurology.

[9] Armstrong MJ, Litvan I, Lang AE, Bak TH, Bhatia KP, Borroni B (2013). Criteria for the diagnosis of corticobasal degeneration. Neurology.

[10] Boeve BF (2011). The multiple phenotypes of corticobasal syndrome and corticobasal degeneration: implications for further study. J Mol Neurosci.

[11] Giorelli M, Losignore NA, Bagnoli J, Difazio P, Zimatore GB (2014). The progression of posterior cortical atrophy to corticobasal syndrome: lumping or splitting neurodegenerative diseases?. Tremor Other Hyperkinet Mov (NY).

[12] Chahine LM, Rebeiz T, Rebeiz JJ, Grossman M, Gross RG (2014). Corticobasal syndrome: five new things. Neurol Clin Pract.

[13] Hsiung GY, Fok A, Feldman HH, Rademakers R, Mackenzie IR (2011). rs5848 polymorphism and serum progranulin level. J Neurol Sci.

[14] Tartaglia MC, Sidhu M, Laluz V, Racine C, Rabinovici GD, Creighton K (2010). Sporadic corticobasal syndrome due to FTLD-TDP. Acta Neuropathol.

